# The Sabbath

**Published:** 1892-05

**Authors:** 


### THE SABBATH.

President Andrews, of Brown University, a clergyman of one of the most straight-laced of the Evangelical denominations, has been lecturing to the students under his charge on the Sunday question. He said that the attitude of Christ showed that the Old Testament Sabbath was to be done away with, and that the first day of the week is not given any prominence in the New Testament as a day of rest, that Christians carried on their work, the same as on any other day down to the time of Constantine. The basis of the Sabbath obligation he said does not consist in the sacredness of any given day, but man needs a certain proportion of time for rest. Puritanism was a dreadfully narrow manifestation of Christianity; and we are suffering from it now. The Sabbath isn't meant to be a kind of mustard plaster, to torment mankind. President Andrews said further, as reported in the Providence *Evening Telegram :* As to the question, "What is the proper observance of Sunday?" I answer : The primary thought is rest. This was the main thought of the Bible. For those who can devote Sunday to spiritual uses it is a duty; but they should not enforce such uses on others. We have no right to prohibit by law any of those who wish to go down the bay on Sunday. I have a theory that the ministers of this city might do an immense amount of good in the summer by instituting Sunday missions on the excursion steamers. As soon as the boat leaves the docks, let a half dozen young people, with good voices, sing several hymns, and then let some enthusiastic young man make a stirring address of not over ten minutes on some practical religious topic.

It would not do to offer prayer, because prayer offered by a Protestant minister would be exceedingly distasteful to many on board, and the sermon should be conducted so that none would be offended. Such service would be very beneficial. All art galleries and free libraries should be open on Sunday, for such marks of progress are not a breach of the Lord's day. The persons who object to this don't know what poverty is ; and just as long as ministers object to such things, they will gain no hold on the laboring people. This is just the class they fail to reach, while on the contrary they were just the ones that Christ did get hold of. On these grounds, I advocate the opening of the World's Fair on Sundays, but not all day. Let it be opened at noon for the rest of the day. Possibly, it would not be well to have the machinery department open; but all the art galleries, and every thing of that nature, should be open, by all means.

